# Intimate Partner Violence Documentation and Awareness in an Urban Emergency Department

**DOI:** 10.7759/cureus.6493

**Published:** 2019-12-28

**Authors:** Janeske Vonkeman, Paul Atkinson, Jacqueline Fraser, Rose McCloskey, Adrian Boyle

**Affiliations:** 1 Family Medicine, Dalhousie University, Saint John, CAN; 2 Emergency Medicine, Saint John Regional Hospital, Saint John, CAN; 3 Emergency Medicine, University of New Brunswick, Saint John, CAN; 4 Emergency Medicine, Addenbrookes Hospital Cambridge University, Cambridge, GBR

**Keywords:** intimate partner violence, domestic violence, emergency department

## Abstract

Background

Domestic violence rates in smaller cities have been reported to be some of the highest in Canada. It is highly likely that the staff at emergency departments (ED) will come in contact with victims of intimate partner violence in their daily practice. The purpose of this study is to better understand current practices for detecting intimate partner violence, staff awareness and knowledge regarding intimate partner violence, and barriers to questioning about intimate partner violence in the ED.

Methods

A standardized retrospective chart review captured domestic violence documentation rates in patients presenting to the ED, and a cross-sectional online survey was distributed to the ED staff.

Results

We found documentation about intimate partner violence in 4.64% of all included patient charts. No documentation was noted in the domestic violence field. Significantly, 16.4% of the ED staff reported never questioning female patients about intimate partner violence; 83.6% enquired when they thought it appropriate, and none asked routinely. None of the staff used a structured screening tool, and 81.8% of the ED staff had not received any formal training. Partner presence was the most common barrier to asking about intimate partner violence, followed by a lack of access to domestic violence management information, and a lack of knowledge regarding intimate partner violence.

Conclusions

Our findings suggest that the current documentation tools are not being properly utilized. Low rates of intimate partner violence documentation in high-risk patients and lack of education indicate that there is a need to improve current practices. In order to improve identification of this important problem, appropriate training and education about intimate partner/domestic violence are required to increase staff comfort as well as knowledge about available community resources for the victims.

## Introduction

There is no generally accepted definition of domestic violence in the medical literature, and there is wide variation in the terms used to describe the phenomenon [1]. Canadian law enforcement categorizes intimate partner violence and abuse into three categories; physical violence, sexual assault, and emotional abuse, which can be committed by a “spouse, ex-spouse, a current or former common-law partner, a current or former girlfriend or boyfriend, or a person in a dating relationship” [2]. Physical assault may include “a threat with a fist or object; being pushed or shoved in a way that could result in injury; being slapped, hit or beaten; being hit or attacked with an object. There may be no obvious physical injuries, or there may be bruises, cuts, broken bones, internal injuries, disfigurement, disablement, and even death.” Domestic violence falls under the umbrella of family violence, which includes three primary victim groups: spouses, children and youth under 18, and seniors over 65. Recently, it has been argued that violence against dating partners falls within the definition of family violence, as this has many similarities to spousal violence [3]. For the purpose of this study, we will use the term intimate partner violence when assessing physical domestic violence against females between the ages of 16 and 64 by their partners. Due to the high rates of intimate partner violence among youth [1], we have chosen to include youth between the ages of 16 and 18 years.

According to Statistics Canada, the police reported 90,300 victims of violence by an intimate partner in 2013; intimate partner violence accounted for 53% of police-reported incidents and spousal violence for the remaining 47% [4]. Physical assault, including pushing, slapping, punching, and face-to-face threat, was involved in the majority of police-reported incidents of family violence [4]. Furthermore, incidents between dating partners have been found to account for over 25% of all violent incidents [5]. Domestic violence has been found to be prevalent among patients presenting with traumatic injuries. The vast majority of domestic violence incidents recorded by the healthcare staff are not recorded by the police [6]. In a retrospective analysis of the American National Trauma Data Bank, the reporting rate for domestic violence was found to be 5.7 cases per 1,000 trauma center discharges (6). In addition, a cross-sectional study in a variety of US emergency departments (EDs) showed that the incidence of acute domestic violence in female patients with a male partner was 11.7% [7]. Intimate partner violence is not only a relevant problem to Canada but also within Atlantic Canada. According to a 2010 statistics Canada report, [3] smaller census metropolitan areas were found to have the highest rate of family violence. For example, the recorded rate in Saint John, New Brunswick was found to be four times the rate recorded in Ottawa, Ontario; Saint John reported 420 victims for every 100,000 people compared to the national average of 294 per 100,000 [3]. It is estimated that before seeking help, women can experience up to 35 episodes of domestic violence [1,8]. While screening all female patients for intimate partner violence is not consistently supported by the literature [9,10], it is generally accepted that clinicians should have a low threshold when querying victims about intimate partner violence and it is beneficial to screen high-risk groups.

Various screening tools for domestic violence are available and, typically, these tools are relatively easy to administer [11]. However, many of these tools have only been evaluated in a limited number of studies and do not have well-established psychometric properties [11,12]. The Partner Violence Screen is a commonly used tool that consists of three questions: 1) “Have you been hit, kicked, punched, or otherwise hurt by someone within the past year? 2) Do you feel safe in your current relationship now? 3) Is there a partner from a previous relationship who is making you feel unsafe now?” [12]. The first question addresses physical violence while the second and third questions address the woman’s perception of safety [13]. Any positive response constitutes a positive screen for intimate partner violence. The Partner Violence Screen has been partially validated in women presenting to urban EDs [13]. In a review of screening tools, the Partner Violence Screen was shown to have a sensitivity of 35-71% and specificity of 80-94% [14].

Even with the availability of various screening tools, there are many barriers to inquiring about domestic violence [14]. Health professionals might not consider domestic violence in patients from higher socioeconomic classes due to the erroneous assumption that domestic violence occurs exclusively in women from lower socioeconomic classes [1]. Many health professionals may feel uncomfortable asking questions about abusive relationships [14]. Although a difficult conversation to initiate, a “significant percentage of women will disclose domestic violence” and are comfortable discussing this with ED physicians and nurses post triage [8]. Furthermore, it is suggested that simply asking a question in itself can be regarded as a meaningful step. Experts suggest that it may reduce the feeling of helplessness experienced by victims and indicate to them that help is available [15]. According to the Society of Obstetricians and Gynecologists of Canada 2005 guidelines, “asking women about violence is not a screening intervention” as victims are not asymptomatic [16]. The guideline suggests that “training of health care providers may reduce barriers to asking about violence”, and that women “often choose to disclose when asked.” [16]. 

The purpose of this study is to better understand current practices for detecting intimate partner violence in an ED in Atlantic Canada. This department is the only level 1 trauma center and the province’s largest tertiary hospital. We wish to determine if patients presenting to the ED are assessed for intimate partner violence and to describe current documentation practices. We will also describe the ED staff awareness and knowledge surrounding intimate partner violence, currently accepted screening questions, and available screening tools. Current barriers to questioning about intimate partner violence in patients presenting to the ED will also be discussed. Finally, we will survey the ED staff to determine if they are willing to implement the brief intimate partner violence screening tool in their daily practice.

## Materials and methods

**Figure 1:**
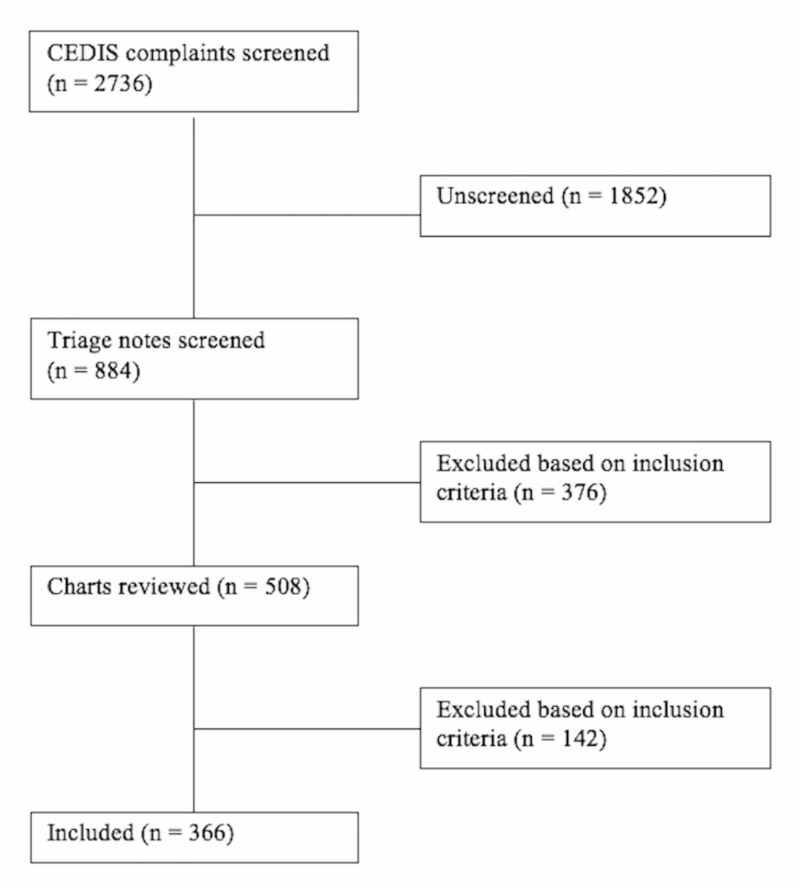
Flow diagram of the health records review CEDIS: Canadian Emergency Department Information System

Phase one of this ambispective study included a standardized health records review (Figure 1). Women aged 16 to 64 years who presented to the ED with injuries that appear to have been caused by intentional violence, such as facial injuries, lacerations, and burns were included (see Appendix for a complete list of included injuries). After receiving approval from the Horizon Health Network Research Ethics Board (2016-2320), we identified target patients using specified search criteria based on the Canadian Emergency Department Information System (CEDIS) presenting complaints list. The electronic database search was followed by a manual application of inclusion and exclusion criteria using the triage note and chart review. Chart review included intimate partner violence case identification and any documentation evaluated. For this study, we excluded patients younger than 16 and over 64 years of age. In addition, female patients with a clearly identifiable non-intimate partner violence-related cause for their injuries, such as motor vehicle accidents or sporting injuries, were excluded. For the purposes of the present study, we did not include males in our initial chart review, though we acknowledge that intimate partner violence is also a serious problem in this population [1]. Data were collected by two researchers to capture domestic violence documentation rates in patients presenting to the ED between January and April 2015. Documentation was recorded in the domestic violence field, a checkbox next to "DV" located in the vital sign section of the patient chart, the nursing triage note, as well as physician/nursing charting.

The second phase consisted of a cross-sectional survey of the ED staff (See Appendix 1). A 17-question survey created specifically for this study by Google Forms was used to assess the ED staff awareness and knowledge of screening tools. The survey was distributed and completed electronically by the ED staff (licensed practical nurses, nurse practitioners, physicians, residents, registered nurses) via staff email lists three times between July and October 2016. Data collected included information pertaining to domestic violence field usage, documentation in patient charts, current questioning habits, correct identification of appropriate intimate partner violence questions, awareness of available screening tools, whose role it is to question patients and whether formal training has been received, barriers faced in the ED, willingness to implement a new screening tool, and whether the staff would find this tool beneficial. Part of this data has been presented at the Canadian Association of Emergency Physician (CAEP) conference in 2017 and published later on [17-19].

## Results

**Figure 2:**
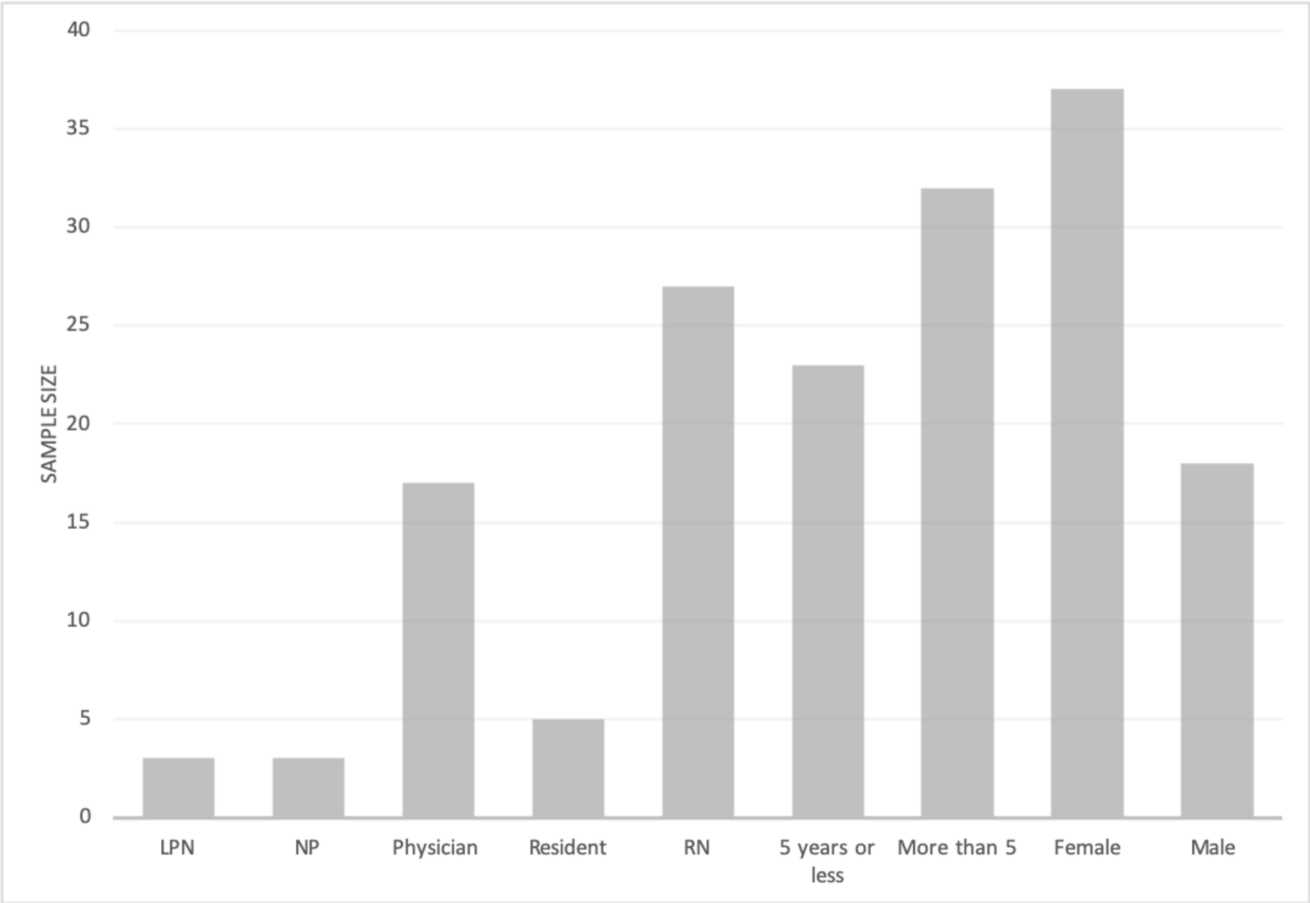
Emergency staff survey demographic information LPN: licensed practical nurse; NP: nurse practitioner; RN: registered nurse

During the health records review, 366 patient charts were analyzed for ED visits between January and April 2015. The online survey had a response rate of 45.9% (n = 55). The demographics of survey respondents are shown in Figure 2.

Current domestic violence documentation practices

**Figure 3:**
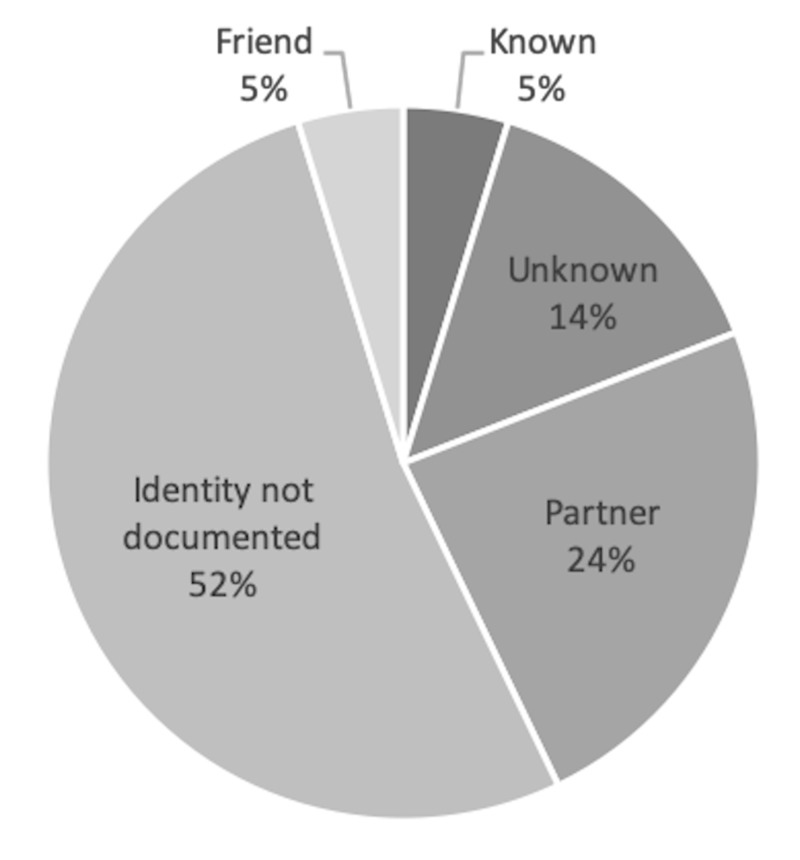
Documented assailant identities of deliberate injuries

**Figure 4:**
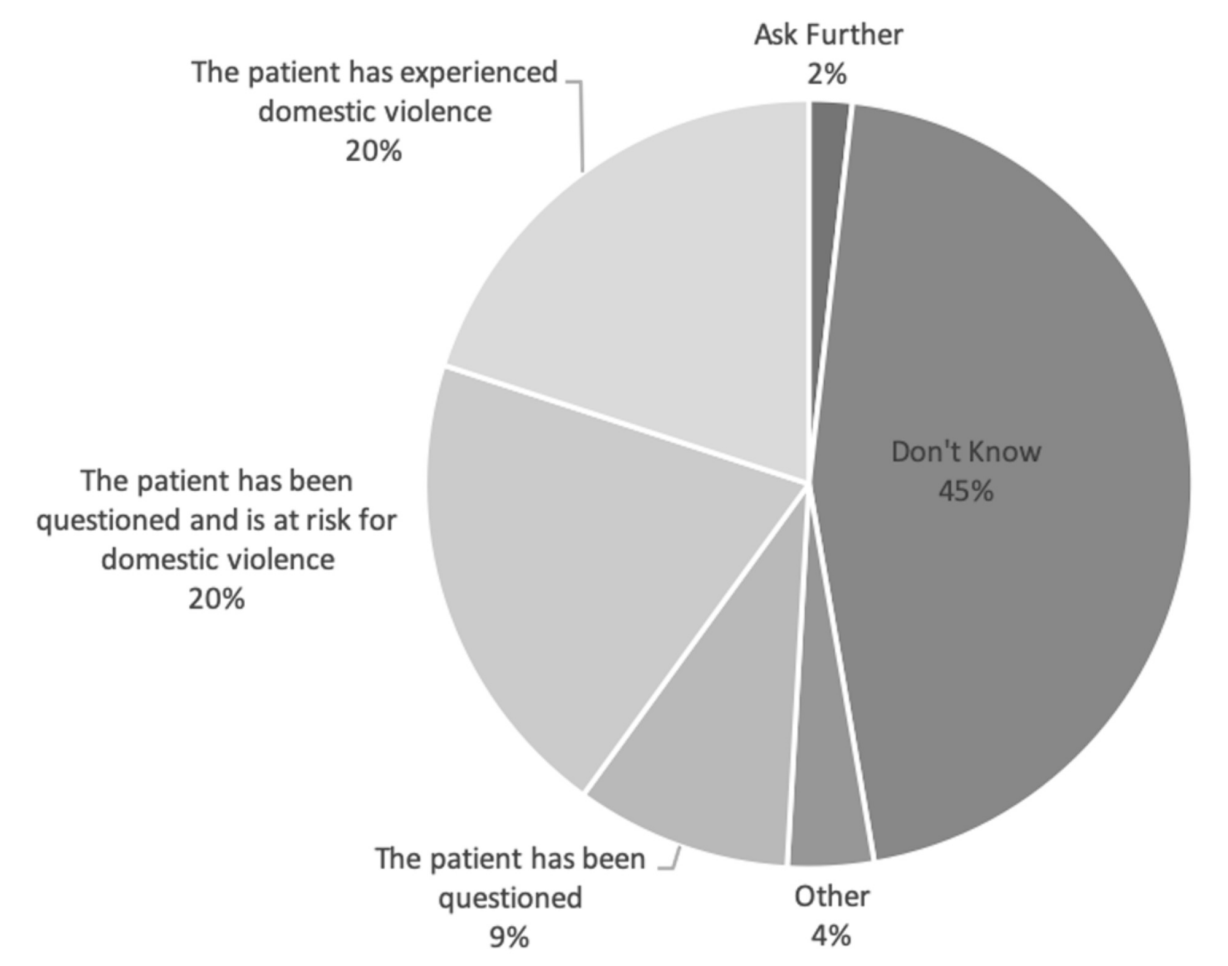
Emergency department staff interpretation of a positive domestic violence field

Overall, we found intimate partner violence documentation in 4.64% of all included patient charts (n = 366). No documentation was noted in the domestic violence field ("DV"). Over half (52.4%) of patients with deliberate injuries had no documentation of assailant identity (Figure 3). With regards to self-reported documentation practices, 16.4% of the ED staff never questioned female patients about intimate partner violence, 83.6% asked when thought appropriate, and none reported asking routinely. None of the staff used a structured screening tool. Of note, 60% of the ED staff documented their questioning, but 92.7% never used the domestic violence field for documentation; 58% of the ED staff could not identify the domestic violence field, and 45.5% of respondents did not know how to interpret the domestic violence field if positive (Figure 4).

ED staff awareness and knowledge of intimate partner violence and available tools

**Figure 5:**
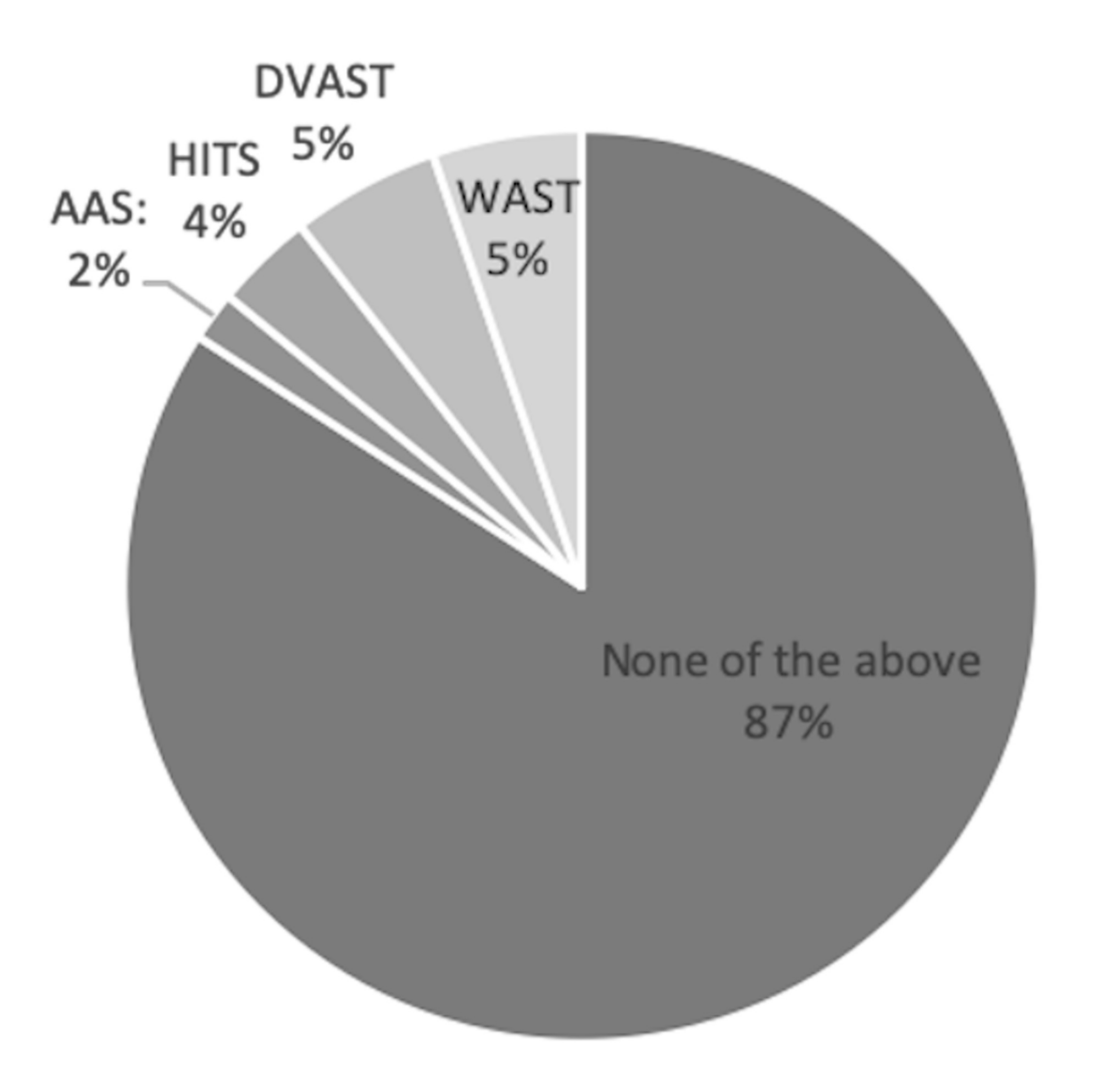
Awareness of screening tools among emergency department staff AAS: abuse assessment screen; HITS: hurt, insult, threaten, and scream; DVAST: domestic violence risk assessment tool; WAST: woman abuse screening tool

When asked to identify recommended questions about intimate partner violence, the staff were more likely to choose appropriate screening questions (75.3%; 95% CI: 69.3-80.6%) compared to questions that are not recommended (23.8%; 95% CI: 19.4-30.7%). However, 87.3% of respondents were not aware of current screening tools (Figure 5). Around half (49.1%) believed that all patients with “typical “injuries (excluding facial injury) should be questioned further about intimate partner violence; 20% believed that all patients with any injury should be questioned, and 16.4% believed that all patients should be questioned. Most (89.1%) also felt that it is both the physician’s and nurse’s role to question patients about intimate partner violence. Significantly, 81.8% of the ED staff had not received any formal training on domestic or intimate partner violence.

Barriers to questioning about intimate partner violence

**Figure 6:**
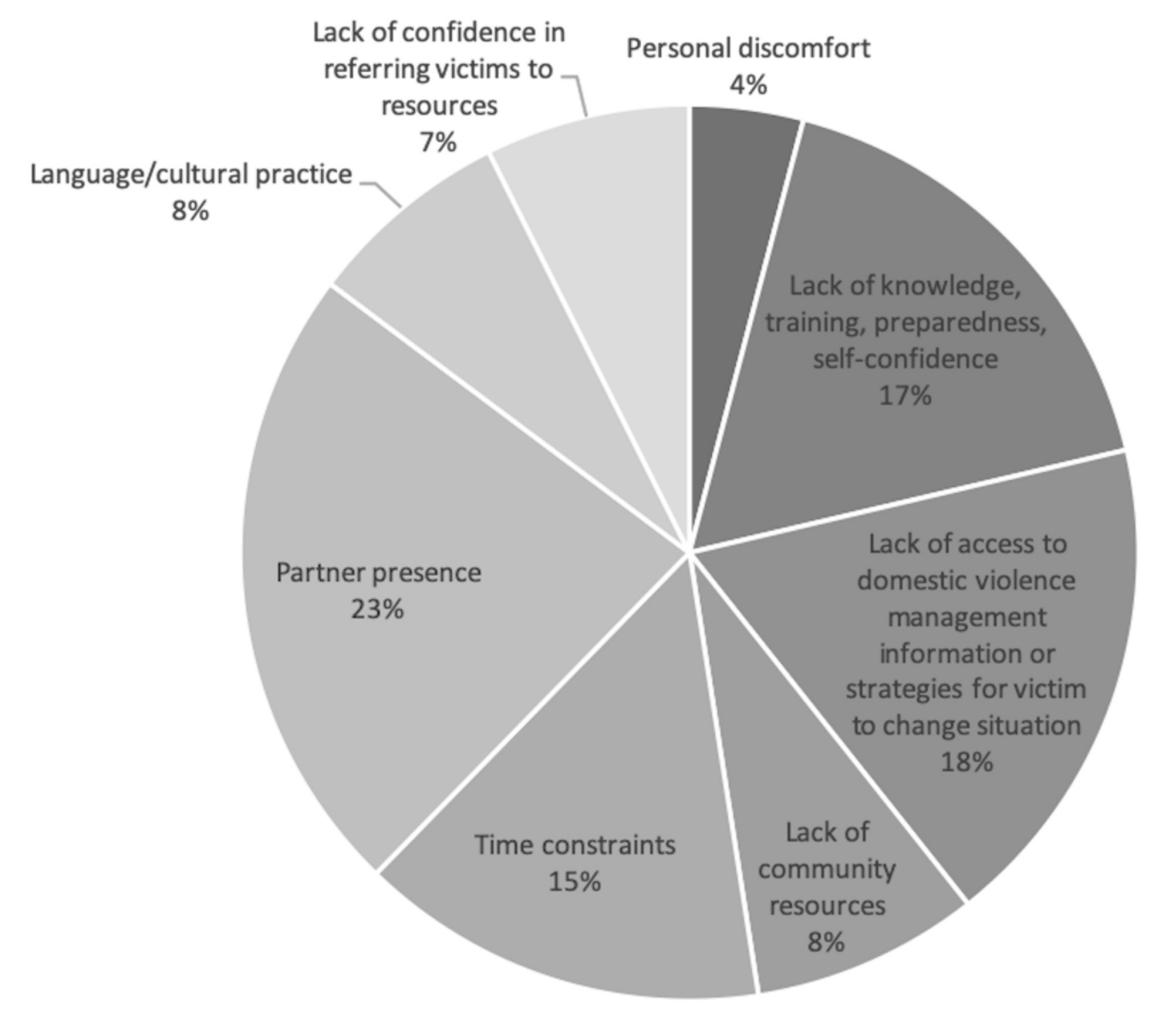
Barriers to intimate partner violence questioning as reported by emergency department staff

Partner presence was the most common reason cited for not asking about intimate partner violence by the ED staff (23.0%). This was followed by a lack of access to domestic violence management information or strategies for the victim to change their situation (18%), lack of knowledge, training, preparedness, and self-confidence (17.2%), and time constraints (14.8%) (Figure 6).

Willingness to implement a brief intimate partner violence screening tool

Almost half (43.6%) of the staff responded that they would be likely or very likely (2.7%) to use an intimate partner violence tool routinely; 7.27% and 3.64% stated their predicted use as unlikely and very unlikely, respectively, while 29.1% were unsure. In addition, 43.6% of the staff thought that the Partner Violence Screen would likely be beneficial in identifying intimate partner violence; 12.7% thought very likely; 1.82% thought unlikely; 1.82% very unlikely, and 40% was unsure.

## Discussion

Our study found low rates of intimate partner violence documentation and that there was no use of an already existing documentation tool. Instances of victims of Intimate partner violence seeking help are common in the ED [5,7]. Implementing routine questioning may increase documentation and identification practices. One review found that routine screening increases the identification of intimate partner violence and that women who are identified are more likely to experience intimate partner violence in the following months [20]. In addition, a Cochrane systematic review and meta-analysis identified six studies, which showed that screening increased the identification of intimate partner violence [9]. While there is a lack of evidence in the literature that screening affects outcomes, a routine inquiry may anyway be justified.

We found that there may be a lack of education and training about intimate partner violence among the ED staff, even though it was felt that it fell within their responsibility as healthcare providers. Similar findings have been reported in the literature. One study found that family medicine physicians felt it was their responsibility to identify and treat intimate partner violence but reported less comfort and decreased likelihood of screening for intimate partner violence, compared to providers within women's health [21]. Another study found that ED staff believed both physicians and nurses have the responsibility to screen for and respond to intimate partner violence [22]. The literature suggests that asking women about domestic violence is deemed acceptable by the majority of women and that women who object are more likely to have experienced recent abuse [23].

In our study, several barriers to intimate partner violence questioning were reported by the ED staff. Similar barriers have been reported elsewhere [14]. Another study on family medicine attendees additionally reported staff turnover, inadequate finance, cultural aspects of violence, helplessness, and lack of competence and qualifications as barriers to active detection of intimate partner violence [24]. A survey of medical residents found that nearly half of the residents felt they received inadequate training, were unprepared to question patients regarding intimate partner violence, and were unsure what to do if the patient disclosed intimate partner violence [25]. Barriers relating to education and awareness of intimate partner violence reported in our study and others suggest the need for raising more awareness on this topic. 

Our study has several limitations. We did not include males in our retrospective chart review sample. Due to time and resource restrictions, we chose to limit the study to females in order to capture intimate partner violence rates in a patient population where it is most prevalent [26]. However, we recognize that intimate partner violence is a serious issue that also affects males, and future research could consider the inclusion of males. Furthermore, the health records review was completed on visits to the ED between January and April of 2015. Future studies could include expanding the review period and thereby increase the sample size and reduce any seasonal bias. With regards to survey data, our survey was sent out via staff email lists on three occasions with a staff response rate of 45.9%. Distributing paper surveys in the ED department and increasing the number of emails are two methods that might increase the response rate. Finally, our reliance on self-reports for information concerning assessment practices, awareness, and knowledge of intimate partner violence may have introduced reporting biases. 

It is highly likely that the ED staff will come across victims of intimate partner violence in their practice. Our findings suggest that the current documentation tool (a domestic violence field) is not being utilized. Furthermore, the low rate of intimate partner violence documentation in high-risk patients indicates that there is a need to improve current practice. Our results indicate that there may be a gap in education about this important problem as revealed by the lack of knowledge surrounding current tools, lack of consensus on who should be questioned, and lack of training. In addition to a lack of awareness, we have also identified several barriers faced by ED staff when questioning patients about intimate partner violence.

In order to improve the identification of intimate partner violence, appropriate training and education about intimate partner violence/domestic violence are required. This would help increase staff comfort and lead to increased awareness of available community resources for management and strategies for victims. Moving forward, we hope to introduce and evaluate an intimate partner violence routine inquiry tool, the Partner Violence Screen, through a knowledge translation education piece. We will assess whether further education, training, and introduction of the tool will improve the identification process and awareness of this important problem in a vulnerable population group. Our findings suggest that ED staff may be receptive to this and will find the introduction of the Partner Violence Screen beneficial in identifying cases of intimate partner violence. We hope that the implementation of this knowledge translation protocol will increase awareness and comfort and improve the identification and documentation process and, ultimately, result in more appropriate care and patient-centered outcomes in this high-risk, vulnerable population group [1,3].

## Conclusions

Our findings suggest that current intimate partner violence documentation tools are not being properly utilized. Low rates of intimate partner violence documentation in high-risk patients and a lack of education among the ED staff indicate that there is a need to improve current practices. In order to improve the identification of this important problem, appropriate training and education about intimate partner violence/domestic violence are required as this will definitely instill awareness among the ED staff about available community resources for victims.

## Appendices

Appendix 1. List of Canadian Emergency Department Information System (CEDIS) presenting complaints selected as high risk for violent causation.

1. ENT

Ear trauma - 056

Facial trauma - 102

Neck trauma - 105

Epistaxis - 151

Nasal trauma 155

2. Gastrointestinal

Anal/rectal trauma - 264

Genitourinary              

Genital Trauma - 310

3. Neurologic

Head injury - 407

4. OB/GYN

Sexual assault - 454

SANE

Pregnancy issues <20 weeks

Pregnancy issues, >20weeks

5. Ophthalmology

Eye trauma - 510

6. Orthopedic

Traumatic back/spine injury - 552

Upper extremity injury - 556

Lower extremity injury - 557

7. Respiratory

Hemoptysis - 655

8. Skin

Bite -701

Abrasion - 703

Laceration/puncture - 704

Burn - 705

Spontaneous bruising - 716

9. Trauma

Major trauma - penetrating - 801

Major trauma - blunt - 802

Isolated chest trauma - penetrating - 803

Isolated chest trauma - blunt - 804

Isolated abdominal trauma - penetrating - 805

Isolated abdominal trauma - blunt - 806

ED Staff Survey

1. Which of the following best describes you?

RN

LPN

Resident

Physician

2. How long have you been practicing in the emergency department (include years practiced outside of Saint John):

1. Please check the box which applies to you:

Female

Male

Other

Prefer not to say

2. How old are you?

1. What do the following abbreviations in the patient record mean?

BP: ____________________

BS: ____________________

DV: ____________________

2. Do you question about Intimate Partner Violence in female patients?

Never

Routinely

When appropriate

3. If you answered “routinely” or “when appropriate” to question 6, do you document whether the patient was questioned about partner violence in the patient chart?

Yes

No

4. If you answered “routinely” or “when appropriate” to question 6, do you use a structured domestic violence screening tool?

Yes:

No

5. If you answered yes to question 8, which screening tool do you use?

6. Do you use the DV field to record questioning?

Yes

No

7. If DV is checked, how do you interpret it?

The patient has been questioned

The patient has been questioned and is at risk for domestic violence

The patient has experienced domestic violence

1. Which of the following screening tools are you aware of? Check all that apply.

HITS: Hurt, Insult, Threaten, and Scream

WAST: Woman Abuse Screening Tool

SAIPV: Screening Assessment for Intimate Partner Violence

PVS: Partner Violence Screen

AAS: Abuse Assessment Screen

DVAST: Domestic Violence Risk Assessment Tool

None

2. Why might you NOTask about for intimate partner violence? Check all that apply.

Personal discomfort with the issue

Lack of knowledge, training, preparedness, self-confidence

Lack of access to domestic violence management information or strategies for victim to change situation

Lack of community resources

Time constraints

Partner presence

Language/cultural practice

Lack of confidence in referring victims to resources

Other:

1. Which of the following questions are recommended for asking about intimate partner violence? Check all that apply.

Have you been hit, kicked, punched, or otherwise hurt by someone within the past year?

Do you feel safe in your current relationship now?

Has your partner ever abused you?

Is there a partner from a previous relationship who is making you feel unsafe now?

Are you a victim of intimate partner violence?”

Has your partner ever hurt or threatened you?

Have you ever been afraid of your partner?

Has your partner ever hit or hurt you?

I am concerned that your partner might be abusing you.

2. Which type of patient should have further questioning?

All patients

All women

All patients with any injury

All women with any injury

All patients with typical injuries: ex. Facial injury, lacerations

All women with typical injuries: ex. Facial injury, lacerations

At discretion of physician

Other: ________________________

3. Have you ever received any formal training on Domestic Violence/Intimate Partner Violence:

Yes

No

We are proposing the introduction of the following 3-question Partner Violence Screen.

1. Have you been hit, kicked, punched, or otherwise hurt by someone within the past year?

2. Do you feel safe in your current relationship now?

3. Is there a partner from a previous relationship who is making you feel unsafe now?

1. How would you rate this tool in terms of the likelihood you will use it routinely?

Very unlikely

Unlikely

Unsure

Likely

Very Likely

2. Do you think this tool will be beneficial in case finding for intimate partner violence?

Very unlikely

Unlikely

Unsure

Likely

Very likely
